# Future Trends in Disability and Its Determinants Among Chinese Community Patients With Anxiety Disorders: Evidence From a 5-Year Follow-Up Study

**DOI:** 10.3389/fpsyt.2021.777236

**Published:** 2021-12-09

**Authors:** Zhaorui Liu, Peijun Li, Huifang Yin, Minghui Li, Jie Yan, Chao Ma, Hua Ding, Qiang Li, Zhengjing Huang, Yongping Yan, Changgui Kou, Mi Hu, Jing Wen, Shulin Chen, Cunxian Jia, Yueqin Huang, Guangming Xu

**Affiliations:** ^1^National Clinical Research Center for Mental Disorders & Key Laboratory of Mental Health, Peking University Sixth Hospital (Institute of Mental Health), Ministry of Health (Peking University), Beijing, China; ^2^Mental Health Center of Tianjin Medical University, Tianjin Anding Hospital, Tianjin, China; ^3^School of Government, Peking University, Beijing, China; ^4^Institute of Social Science Survey, Peking University, Beijing, China; ^5^National Center for Chronic and Noncommunicable Disease Control and Prevention, Beijing, China; ^6^Department of Epidemiology, The Fourth Military Medical University, Xi'an, China; ^7^Department of Epidemiology and Biostatistics, School of Public Health, Jilin University, Changchun, China; ^8^Xiangya School of Public Health, Changsha, China; ^9^Department of Epidemiology and Health Statistics, School of Public Health and Management at Ningxia Medical University, Yinchuan, China; ^10^Department of Psychological and Behavior Science, Zhejiang University, Hangzhou, China; ^11^Department of Epidemiology, School of Public Health, Cheeloo College of Medicine, Shandong University & Shandong University Center for Suicide Prevention Research, Jinan, China

**Keywords:** anxiety disorders, disability, future trends, determinants, follow-up study

## Abstract

**Background:** Anxiety disorders (ADs) are a group of disorders with a high disability rate and bring a huge social burden. In China, information on future trends in the disability among community ADs patients and its determinants are rare. The objectives of this study are to describe the future trends in the disability among ADs patients living in community and to investigate the determinants of the disability.

**Methods:** Participants diagnosed with 12-month ADs in the China Mental Health Survey (CMHS) were followed up by telephone from April to June 2018 to assess the future trends in the disability in a 5-year interval using the World Health Organization's Disability Assessment Schedule 2.0. The disability rate was reported and its determinants were analyzed by complex sample design multivariate logistic regression.

**Results:** Totally 271 patients were interviewed by telephone and 33 informants finished proxy interviews. The disability rates were 45.9% and 14.3% among ADs patients at baseline and during the follow-up. Patients with general anxiety disorder (GAD) or agoraphobia with/without panic disorder (AGP) had the lower decrease and higher disability during the follow-up than patients with other subtypes. Patients aged in middle age (aged 40–49 years old, OR = 11.12, 95% CI: 4.16–29.72), having disability at baseline (OR = 7.18, 95% CI: 1.37–37.73), having comorbidity with three or more physical diseases (OR = 9.27, 95% CI: 2.48–34.71), and having comorbidity with other mental disorders (OR = 3.97, 95% CI: 1.13–13.96) had higher disability during the follow-up.

**Conclusions:** The disability rate tends to decrease among ADs patients living in communities. Treatment priority should be given for ADs patients with disability and those in middle age. Treatments for the comorbidity of other mental disorders or physical diseases should be considered when treating anxiety.

## Introduction

Anxiety disorders (ADs) are a group of psychological conditions characterized by an intense sense of anxiety and fear (1). The results of the World Mental Health Survey Initiative showed that the lifetime prevalence of ADs was from 4.8 to 31.0% (2) and 12-month prevalence for ADs was from 2.4 to 18.2% globally (3). The China Mental Health Survey (CMHS) reported ADs are the most prevalent mental disorders with 5.0 and 7.6% for 12-month and lifetime prevalence among representative community Chinese adults (4). Besides its high prevalence, ADs also contribute tremendously on the disease burden (5). Findings from the Global Burden of Disease (GBD) study showed that ADs account for 14.6% disability adjusted life years in 2017 (6). An analysis of the economic burden of mental disorders in China from 2005 to 2013 estimated that ADs contributed to about 30% of total costs of all mental disorders (7).

Disability is defined by as functioning restrictions or activity limitations in multiple dimensions of life based on the International Classification of Functioning, Disability and Health Framework (8, 9). According to this definition, ADs could lead to function impairments. Among ADs patients, the symptoms of fear and anxiety are marked persistent, which are associated with disabilities in social, occupational, or other important areas of functioning (10). Results from the World Mental Health Surveys showed clearly evidences of the disability among ADs patients (11). Compared with current estimations of disability, the future trends in the disability of ADs patients might have a more important implications from a public mental health view. The importance could be explained at two levels. First, as mental health services have continuously struggled for adequate funding allocation, using disability as an outcome variable makes a strong advocacy case toward investing in mental health (12). Second, resource allocation decisions should continuously require information on disability which has been considered a reliable indicator to set priorities within the field of mental health (13).

The future trends in the disability among ADs patients are affected by many factors. A Dutch longitudinal study over 4 years of time evaluated the long-term disability of ADs patients and found the future trends in the severities of disability might be various across different subtypes of ADs. The long-term disability was the highest in participants with social anxiety disorder and multiple ADs (comorbidity with other anxiety disorders) and was the lowest in panic disorder with agoraphobia and panic disorder without agoraphobia. General anxiety disorder was in the middle position (14). This longitudinal study also indicated that anxiety arousal and avoidance behaviors were major predictors for long-term disability (14). Other factors related to disability were proposed to be the female gender, increased age, low family income and no medical insurance (15, 16). However, current evidence on the future trends in the disability are not sufficient. Some studies focus on specific anxiety disorder rather than the whole category of anxiety disorders (17, 18). And to our knowledge, few study about the future trends in the disability among ADs patients and its determinants has been conducted in China. Therefore, a follow-up study about the future trends in the disability was conducted after 5 years from the time when the CMHS was finished. The aims of the current study were to describe the future trends in disability in ADs patients living in community and to investigate the predictors of the trends. Findings from the study could provide evidence for the developments of health policy strategies, and the adjustments of treatment programs for ADs patients in China.

## Materials and Methods

### Samples and Procedures

The detailed study design of the CMHS had been published elsewhere (19). In brief, the CMHS was a national representative epidemiological survey on mental disorders and mental health services in China from 2012 to 2015. Totally 28,140 community adults aged 18 years or over were interviewed by trained lay interviewers using the Composite International Diagnostic Interview 3.0 (CIDI-3.0) in the CMHS. From April to June 2018, a follow up study by telephone using computer assisted telephone interview (CATI) mode was carried out among all participants who had finished CIDI interviews. The sample of this study included all individuals who were diagnosed with 12-month ADs in the CMHS.

Fifty interviewers attended 2-day trainings for the use of CATI, the procedure of the study, skills in telephone interview, and the content of questionnaire, and the quality control methods, and passed the final examinations of the training. Interviewers were blinded with the diagnoses of all participants during the survey. Totally four supervisors were responsible for the interviewing standards of the interviewers. Confirmations to the attendances of the CMHS were made before main interviews. Interviews would be continued if the sample met any of the two criteria. One criterion was that at least one name of the household member was correctly matched with baseline database. The other criterion was the address of the sample was correct, and the participants or their informants remembered the attentions to the CMHS. Participants of the CMHS had the priorities to answer the questionnaires of the follow-up study. But proxy interviews by informants were allowed if the participants had one of the following situations. First, participants refused to be interviewed for three times. Second, participants were unable to be contacted in six attempts at different time and date. Third, participants were unable to be interviewed due to severe physical diseases or mental disorders, hospitalizations, or no telephone after house-moving. The informant should be 18 years old or over and know the situation of the participant very well. All participants and informants were asked for oral informed consents from the telephone. The process of the consents was recorded to audio files.

An independent team consisted of three supervisors and ten staff conducted three types of quality control, including data checks, audio checks, and monitoring checks. The CATI system made it easier to achieve para data during the interviews. Data checks were carried out daily for all interviews to detect systematic errors and their distribution patterns, mainly focusing on the non-response, interruption of the interviews, and times of contact attempts. Audio checks were conducted for about 10% of respondents to identify problems of the interviewers, such as mistakes during the data entering, inaccurate information of the informants, insufficient probing, and irregular behaviors when asking a question. Monitoring checks were made for at least three telephone conversations of each interviewer to confirm if the interviewers had proper behaviors during the interviews. The study has been performed in accordance with the ethical standards laid down in the 1964 Declaration of Helsinki and its later amendments. It was approved by the Ethical Committee of Tianjin Anding Hospital and the Sixth Hospital of Peking University. All participants and informants were provided with oral informed consents prior to their participation in the study, and the process of the consents was recorded to audio files.

### Measurements

#### Diagnose of Anxiety Disorders

ADs were diagnosed using CIDI-3.0, a fully structured interview instrument administered by trained lay interviewers. CIDI has been proved its satisfied validity and was used in China (4, 20) and many other countries for epidemiological surveys (21–23). The 12-month diagnosis were made for panic disorder (PD), agoraphobia with/without panic disorder (AGP), specific phobia (SP), social phobia (SO), obsessive compulsive disorder (OCD), general anxiety disorder (GAD), and not otherwise specified anxiety disorder (NOS) according to the criteria and definition of the Diagnostic and Statistical Manual of Mental Disorders, Fourth Edition (DSM-IV) (24).

#### Assessment for Disability

The World Health Organization's Disability Assessment Schedule 2.0 (WHODAS-2.0) was used to assess the disability in the past 30 days from the interviews (25). The Chinese version of WHODAS-2.0 has been confirmed with good reliability and validity. WHODAS-2.0 includes six domains: (1) cognition—understanding and communicating; (2) mobility—moving and getting around; (3) self-care—attending to one's hygiene, dressing, eating, and staying alone; (4) getting along—interacting with other people; (5) life activities—domestic responsibilities, leisure, work, and school; (6) participation—joining in community activities, participating in society. At baseline detailed questions in each domain were asked before an overall estimation of the impairments of the domain. But during the follow up, the overall estimations were asked first, and detailed questions in a domain would be skipped if the respondents reported no impairment of the domain. Reasons of the change were to reduce the interview length and increase the response rate during the telephone interviews.

To increase the comparability between the assessments at baseline and follow up, the global disability scores were calculated based on the inquiry method during the follow up. In the baseline database, the original answers for detailed questions in a domain were recoded to zero if the respondent reported no impairment of the domain. Given the distributional properties of the changes, the global disability score was dichotomized at the 90th percentile to indicate the presence or absence of disability. This modifications to the WHODAS-2.0 has been reported as a valid measurement of global disability (26).

#### Potential Predictors

All information of potential predictors was collected at baseline. Five sociodemographic factors were collected and categorized, including age (18–39, 40–49, 50–59, 60 years, and over), gender (female or male), residential area (urban or rural), educational level (literate or below primary school, primary school, junior high school, senior high school, and college or university and above), and marital status (married, never married, separated, or divorced). Information of chronic physical diseases was collected based on self-reports, including heart disease, high blood pressure, asthma, chronic lung disease, tuberculosis, diabetes, stroke, stomach ulcers or intestinal ulcers, rheumatic fever or arthritis, and chronic headaches. Three categories, including no physical disease, one or two physical diseases, and three physical diseases and over were defined by using this information. Comorbidity of other mental disorders, including depressive disorders, bipolar disorders, drug use disorders, alcohol use disorders, was made by the CIDI-3.0 and divided into no comorbidity with other mental disorders, and comorbid at least one mental disorder. Data of lifetime and 12-month treatments using mental health services among ADs patients was also included as potential predictors.

#### Contact Information

Contact information both at baseline and follow up was collected for the tracing purposes, including the address, any plans of moving to a new house, contact information of the participants and informants.

#### Statistical Analysis

New weights were generated for the database of the follow up in accordance with the weights in the CMHS database. The methods of weighting were similar as the way in the CMHS, which has been published elsewhere (24). The main difference between the CMHS and the follow up study in the weighting was the way to generate non-response weights, which were also adjusted across different population based on the diagnoses of mental disorders.

Disability rate among ADs patients were presented in this paper. Frequencies and chi-square (χ^2^) tests were used for descriptive analysis and comparisons of rates in different categories. Taylor series linearization method was used to estimate standard errors. Complex sample data logistic regression was performed to analyze the effect of baseline disability and other potential predictors on the disability during the follow up. A significance level of α = 0.05 was used throughout the analysis. Statistical analyses were performed using SAS 9.4.

## Results

### Study Profile and Sample Demographic Characteristics

In the CMHS, 1,155 12-month ADs were diagnosed. During the follow up study, interviewers made attempts to contact persons who had valid telephone numbers of themselves or their informants. Finally, 304 individuals with 12-month ADs at baseline were interviewed with the response rate of 26.3%. Totally 271 (89.1%) persons were the participants in the CMHS and answered the questionnaires themselves, and the rest of 33 (10.9%) persons were informants who finished proxy interviews. In terms of demographic characteristics of 304 patients with 12-month ADs at baseline during the CMHS, there were 124 females (40.8%) and 180 males (59.2%), and the average age was 50.9 ± 13.0 years old. Among these patients, 120 persons (39.5%) were from urban areas and the rest (60.5%) were from rural areas. Only 19(6.3%) persons have ever received treatments during their life, and 12(3.9%) received treatment during the past 12 months. About 0.2% of the interviews failed to pass the data check and were interviewed again. All interviews passed the audio checks and monitoring checks.

### Disability at Baseline and Future Trends in the Disability

Table 1 showed the disability at baseline and future trends in the disability among ADs patients and patients with each subtype of ADs. In total, 153 of the 304 ADs patients met the criteria of disability at baseline, with the disability rate of 45.9% (95% CI: 38.4–53.5%). Five years later, 71 ADs patients met the criteria of the disability at the follow up, with the disability rate of 14.3% (95% CI: 9.5–19.1%). The disability of patients with each subtype of ADs trended to decrease during the follow-up compared with the time at baseline. However, the future trends of disability varied across different subtypes of ADs. Patients with SO had the highest relative decrease rate (82.4%), followed by patients with PD (75.5%). Patients with GAD had the lowest relative decrease rate (19.4%), followed by AGP (43.6%). The relative decrease rates of patients with SP, NOS and OCD were from 65.7 to 69.8%. During the follow-up, patients with AGP or GAD had higher disability than other subtypes, those with OCD or SO had lower disability than other subtypes. The disability rates of patients with SP, PD and NOS were in the middle.

**Table 1:** The weighted disability rate at baseline and follow-up in ADs patients.

**Subtype of ADs**	***N***	**Weighted disability rate**			
		**Baseline**		**Follow-up**	
		**%**	**95% CI**	**%**	**95% CI**
Panic disorder	28	66.9	47.5–86.3	16.4	0–42.4
General anxiety disorder	28	40.8	15.9–65.8	21.4	0–43.5
Specific phobia	147	51.9	38.4–65.3	17.8	8.0–27.7
Social phobia	29	63.3	39.7–86.8	11.1	0–22.6
Agoraphobia with/without panic disorder	22	81.7	58.5–100.0	46.1	19.3–72.8
Obsessive compulsive disorder	109	44.1	32.6–55.5	13.3	4.4–22.2
NOS anxiety disorders	19	46.6	27.0–66.2	14.5	0–31.8
Any anxiety disorders	304	45.9	38.4–53.5	14.3	9.5–19.1

The differences in the distribution of disability rates during the follow up among ADs patients are shown in Table 2. Patients in youngest age group had the lowest disability rate (*p* < 0.001). Patients with lower education level had higher disability than other groups did (*p* = 0.001). Higher disability was present in patients with physical diseases (*p* < 0.001), or other mental disorders (*p* < 0.001). Patients with disability at baseline had higher disability rate than those without disability (*p* < 0.001). There was no statistical difference among patients with different gender (*p* = 0.93) and residence areas (*p* = 0.30). No statistical difference was found between those who sought treatments during their lifetime (*p* = 0.22) or during the past 12 months (*p* = 0.09) and those who did not.

**Table 2:** The disability rates in different categories of demographic and clinical characteristics status and chi-square test results.

**Variables**		**Frequency *n* (%)**	**Disability rates (%)**	**95% CI**	***χ^2^***	***P***
Age in groups	18–39	59 (19.4)	2.6	0.1–5.0	32.3	<0.001
	40–49	93 (30.6)	31.7	16.4–47.0		
	50–59	77 (19.4)	7.1	2.1–12.1		
	≥60	75 (24.7)	20.7	8.0–33.4		
Gender	Female	124 (40.8)	14.6	5.2–24.0	0.0	0.93
	Male	180 (59.2)	14.0	8.1–20.0		
Education level	Junior high school	96 (31.6)	12.4	2.3–22.5	19.6	0.001
	Literate or below primary school	93 (30.6)	27.5	13.7–41.3		
	Primary school	66 (21.7)	10.5	4.8–16.2		
	Senior high school	31 (10.2)	4.4	0–9.5		
	college or university and above	17 (5.9)	0.5	0–1.5		
Marital status	Married	267 (87.8)	15.1	9.2–21.0	5.5	0.065
	Separated/Divorced	16 (5.3)	4.4	0–10.4		
	Never married	21 (6.9)	24.6	9.4–39.9		
Residence area	Urban	120 (39.5)	11.7	3.8–19.7	1.1	0.303
	Rural	184 (60.5)	17.1	11.9–22.2		
Number of physical diseases	No physical disease	83 (27.3)	3.2	1.0–5.4	20.6	<0.001
	1–2 physical diseases	127 (41.8)	17.0	9.6–24.4		
	3 and more physical diseases	94 (30.9)	24.4	12.3–36.6		
Number of other mental disorder	0	201 (66.1)	19.0	8.9–29.1	6.2	0.01
	≥1	103 (33.9)	49.8	25.2–74.5		
WHODAS at baseline	With disability	153 (45.9)	26.9	17.4–36.5	14.4	<0.001
	No disability	151 (54.1)	3.2	0–7.4		
Lifetime treatment for ADs	No	285 (93.7)	13.8	8.8–18.7	1.5	0.223
	Yes	19 (6.3)	27.2	0.5–53.9		
12-month treatment for ADs	No	292 (96.1)	13.6	8.7–18.5	2.8	0.093
	Yes	12 (3.9)	39.6	0–81.6		

### Determinants of the Future Trends in the Disability

Results from a multivariate logistic regression to test the effects of baseline disability and other potential determinants on the disability during the follow up are shown in Table 3. After controlling for the status of respondents, patients with middle age, suffering from other mental disorders, comorbid with three or more physical diseases, and having disability at baseline were the determinants of higher disability during the follow-up (*p* < 0.05).

**Table 3:** Odds ratios of determinants in anxiety disorders, corresponding 95% confidence intervals (CI), and *p*-values obtained by multiple logistic regression models with disability as outcome variable.

	**Variables**	**Multivariate logistic regression**			
		***B***	***S.E*.**	**OR (95% CI)**	***P***
Age groups	18–39	–	–	1	–
	40–49	1.73	0.31	11.12 (4.16–29.72)	<0.001^*^
	50–59	−1.03	0.42	0.70 (0.24–2.07)	0.02^*^
	≥60	−0.01	0.41	1.95 (0.64–5.93)	0.98
Gender	Male	–	–	1	–
	Female	−0.11	0.23	0.80 (0.32–2.03)	0.63
Educational level	Junior high school	–	–	1	–
	Literate or below primary school	1.99	0.58	2.99 (0.94–9.56)	0.001^*^
	Primary school	0.53	0.51	0.70 (0.16–3.12)	0.31
	Senior high school	−0.07	0.78	0.38 (0.05–2.97)	0.93
	college or university and above	−3.34	1.35	0.01 (0.003–0.504)	0.02
Marital status	Married	–	–	1	–
	Separated/Divorced	0.45	0.48	2.66 (0.70–10.05)	0.35
	Never married	0.07	0.50	1.82 (0.44–7.47)	0.88
Residence area	Urban	–	–	1	–
	Rural	0.16	0.27	1.37 (0.47–4.04)	0.56
Number of physical diseases	No physical disease	–	–	1	–
	1–2 physical diseases	−0.01	0.31	2.99 (0.80–11.19)	0.24
	3 and more physical diseases	1.12	0.32	9.27 (2.48–34.71)	<0.001^*^
Number of other mental disorder	0	–	–	1	–
	≥1	0.45	0.28	3.97 (1.13–13.96)	0.03^*^
WHODAS category at baseline	No disability	–	–	1	–
	With disability	0.99	0.41	7.18 (1.37–37.73)	0.02^*^
Lifetime treatment for ADs	No	–	–	–	–
	Yes	−0.41	0.42	0.44 (0.08–2.37)	0.33

*OR, odds ratio; CI, Confidence interval*.

* *p < 0.05*.

## Discussion

The study is the first study to explore the future trends in disability and its determinants in patients with anxiety disorders living in Chinese communities, where the anxiety disorders are highly prevalent (4). The main findings of this study were although about 45.9% of ADs patients had disability at baseline, less patients had disability during the follow-up, and the future trends of the disability varied across different subtypes of anxiety disorders. This study also found a number of factors, including middle age, having disability at baseline, having comorbidity with physical diseases or other mental disorders, predicted the higher disability rate in the future among ADs patients. These findings could help to understand the natural history of ADs patients living in communities, and to evaluate the prognosis of the diseases without any external interventions. Results from this study also can provide policy makers useful information to assess if the current mental health care system can meet the needs, to identify any barriers to mental health services, and to allocate resources to improve care for these patients.

The decrease of disability during the follow-up implies that even without any intended interventions, the conditions of ADs patients living in communities tended to be better than the time when they were diagnosed as 12-month patients. This finding is consistent with a study carried out among adolescents with anxiety disorders, in which 80% of the patients remitted from the disease (27). The recovery might be explained by the fact that anxiety disorders have a strong tendency to naturally wax and wane over time (28). However, there were about 14.3% of ADs patients who still had disability, which suggests that if any effective treatments of interventions could be provided to patients, their disability should improve. Previous researches have proved that early prevention promised to be very effective by off-setting functional impairments associated with anxiety disorders (10), and effective treatments or interventions would result in substantial burden averted (5).

Middle age, especially from 40 to 49 years old, was found to predict the disability of ADs patients during the follow-up. People in this age group usually have more pressure from their families and works (5) and therefore their anxiety are more likely difficult to relief, which may lead to persistent disability. This result suggests that patients with middle age should be key population for treatments of ADs.

Patients with disability at baseline predicted the disability during the follow-up. This finding implies that more attention should be paid to improve the functions of AD patients during the treatment process. There are some evidences showing that the disability of ADs patients could still be present even after the remissions of the disease (29). It may take more time for the recovery from functional limitations than the remission of symptoms do (30). Findings from this study showed there was no difference of the disability at the follow-up between patients received treatments and those without. Therefore, other than providing timely treatments for disabled ADs patients to relieve their symptoms, ongoing treatments should be provided for them to improve their functions.

Comorbidity with other mental disorders also should be considered during the treatments, as findings from this study indicated that it was one of the independent determinants of the disability of ADs patients during the follow-up. Many researchers have reported that comorbidity strongly aggravated the disability of patients (30). Amongst ADs patients, the presence of comorbid other mental disorders, such as depression, indicates a more chronic course with worse prognosis (31), which may increase the difficulty of treatments and the possibility of recurrence (31, 32). Another finding from the study was the comorbidity of physical diseases predicted the disability at the follow-up. This result implied that comorbidity strongly aggravated the disability, which has been confirm by other studies (30). Physical diseases might lead to poor self-care of ADs patients, and even in some cases, physical diseases would lead to the aggravation of anxiety symptoms (33). Comorbidity of mental-physical disorders was associated with more severe role impairment (34) and the presence of comorbidities increased the difficulty of clinical and community treatments, which lead to high levels of disability after 5 years of follow-up. This finding suggests that in addition to treat anxiety disorders, physical diseases should also be dealt with as soon as possible. That can effectively reduce the disability of patients (35).

In line with previous studies, this study showed the disability rate at the follow-up varied in different specific ADs (14). Individuals with AGP or GAD had higher disability than other subtypes, and those with OCD or SO had lower disability than other subtypes. The disability rates of patients with SP, PD and NOS were in the middle. AGP is more associated with a chronic course than other anxiety disorders and the chronic course is associated with more disability (36). Besides, in patients with AGP, the symptom of agoraphobic avoidance makes the largest contribution to the disability among all types of ADs (31). Consistent with a previous study (37), GAD patients had higher disability during the follow-up. This can be explained that medical co-morbidities were particularly high among individuals with GAD (37), which brought the higher disease burden. Results of lower disability rate among PD patients was consistent with other studies (30, 37–39). This may be because PD patients tend to have higher recovery rates and lower recurrence rates than those with other types of ADs (40, 41). Similar with the finding in a previous study (42), this paper reported that the disability rate of OCD patients had the lower disability among all types of ADs. OCD patients may be restricted in their work (42) and therefore are more likely to seek professional treatments. Although it is difficult to cure OCD patients, patients still can benefit from the treatment and have significant improvement of their symptoms (43). That leads to a lower level of disability. It should be noted that our findings are different from the Dutch study, which showed that the long-term disability was most prevalent in participants with SO and lowest in AGP and PD (14). The differences might be due to the differences between two populations. Chinese patients may have special patterns regarding the disability. More researches are needed in this field in the future.

The results of this study should be interpreted taking into considerations of several limitations. The number of patients followed up in this study was relatively small, which may be biased to some extent in the representativeness of the sample. But the application of weighting during the data analysis might reduce the impact of the low response rate. At baseline, the disability was measured for the time period of past 30 days, whereas mental disorders were diagnosed based on the symptoms during the past 12 months, which may lead to over-estimations of the disability at baseline. During the follow-up, the skipping strategy was applied and informants were allowed to report the disability status of ADs patients. That may contribute to the under-estimations of the disability during the follow-up. However, considering the difficulties during the implementations of an interview with CATI design, those strategies might be the best way to increase the response rate. There is a methodological weakness when using the disability during the past month at follow-up as the only indicator to reflect the prognosis of ADs, as it cannot represent trends of the disability during the whole 5 years. More measurements should be carried out during the 5 years in the future. Moreover, the assessment of disability was made face-by-face at baseline, while the disability was measured by telephone. The consistency of the two methods should be evaluated in future studies.

## Conclusions

The future trends of the disability presented decrease among ADs patients living in communities. Patients with GAD or AGP had the lower decrease and higher disability during the follow-up than patients with other subtypes, which highlights the treatments needs of these patients. Treatment priority should be given for ADs patients with disability and those in middle age. Treatments for physical diseases or other mental disorders among ADs patients also should be taken in consideration when treating the symptoms of anxiety. The findings of this study had several implications for health policy and reinforce the needs for investment in mental health. In terms of public health agenda, particular attention should be paid to factors that have strong relationship with the future trends of the disability. Taken together, the identification of the specific factors associated with the disability may suggest new directions to improve treatment interventions and may facilitate more appropriate allocation of scarce health care to affected individuals.

## Data Availability Statement

The datasets presented in this article are not readily available because the data of CMHS belong to the National Health Commission (Ministry of Health) of People's Republic of China and are not available for sharing. Requests to access the datasets should be directed to Yueqin Huang, huangyq@bjmu.edu.cn.

## Author Contributions

YH and GX designed the study and obtained the funding for the study with the collaboration of QL, CM, ZH, YY, CK, MH, JW, SC, and CJ. JY and HD acted as research coordinators and were responsible for data collection and quality control. PL, ML, and CM did the analyses with supervision by ZL and HY. ZL and PL wrote the first draft. All authors reviewed the report, provided further contributions and suggestions, and read and approved the final report.

## Funding

This study was supported by the National Key Research and Development Program of China (2017YFC0907800 and 2017YFC0907801). The funders had no role in study design, data collection and analysis, decision to publish, or preparation of the manuscript.

## Conflict of Interest

The authors declare that the research was conducted in the absence of any commercial or financial relationships that could be construed as a potential conflict of interest. The reviewer LS declared a shared affiliation with one of the authors CJ, to the handling editor at the time of the review.

## Publisher's Note

All claims expressed in this article are solely those of the authors and do not necessarily represent those of their affiliated organizations, or those of the publisher, the editors and the reviewers. Any product that may be evaluated in this article, or claim that may be made by its manufacturer, is not guaranteed or endorsed by the publisher.

## Abbreviations

ADs: anxiety disorders
CMHS: China mental health survey
GBD: Global burden of disease
CIDI-3.0: the composite international diagnostic interview-3.0
CATI: computer assisted telephone interview
PD: panic disorder
AGP: agoraphobia with/without panic disorder ()
SP: specific phobia
SO: social phobia
OCD: obsessive compulsive disorder
GAD: general anxiety disorder
NOS: not otherwise specified
DSM-IV: The diagnostic and statistical manual of mental disorders, fourth edition
WHODAS-2.0: World health organization's disability assessment schedule II.
